# Cinnamic acid and magnetized water: A novel nutritional strategy to enhance lamb productivity and meat quality

**DOI:** 10.1016/j.vas.2026.100890

**Published:** 2026-10-05

**Authors:** Mohsen Kazemi, Reza Valizadeh, Abbas Khashei-Siuki

**Affiliations:** a Department of Animal Science, Faculty of Agriculture and Animal Science, University of Torbat-e Jam, Torbat-e Jam, Iran; b Department of Animal Science, Faculty of Agriculture, Ferdowsi University of Mashhad, Mashhad, Iran; c Department of Economic and Water Engineering Sciences, Faculty of Agriculture, University of Torbat Heydariyeh, Torbat Heydariyeh, Iran

**Keywords:** Cinnamic acid, Magnetized water, Lamb performance, Antioxidant status, Meat quality

## Abstract

This study evaluated dietary cinnamic acid (CA; 0 or 400 mg/kg DM) and magnetized water (MW; tap water, 4000, or 6000 Gauss) on growth performance, nutrient digestibility, ruminal fermentation, blood metabolites, antioxidant status, and meat quality in fattening lambs. Thirty-six male Shal lambs were assigned to six treatments in a 2 × 3 factorial design for 56 days. CA improved final body weight, average daily gain, and dry matter intake (*P* < 0.05). Total-tract digestibility of dry matter, organic matter, crude protein, and neutral detergent fiber increased with both CA and MW, with the greatest responses for CA plus 6000-Gauss MW (*P* < 0.05). CA reduced the acetate-to-propionate ratio (*P* < 0.001) and improved serum lipid profiles by decreasing cholesterol and low-density lipoprotein while increasing high-density lipoprotein. Both CA and MW boosted antioxidant defenses by increasing superoxide dismutase and total antioxidant capacity while reducing malondialdehyde (*P* < 0.05). CA also increased glutathione peroxidase and reduced serum tumor necrosis factor-α (*P* < 0.001). Meat quality improved with both treatments, as evidenced by lower pH, greater water-holding capacity, and reduced lipid oxidation, alongside enhanced color traits (L*, a*, b*, Chroma, and Hue), although the effects of MW on L* and a* were not significant alone (*P* > 0.05). Significant CA × MW interactions were detected for serum lipid fractions, meat redness (a*), and Chroma (*P* < 0.05). Overall, combining CA with 6000-Gauss magnetized water resulted in favorable responses in growth performance, nutrient digestibility, antioxidant status, and meat quality under the tested conditions.

## Highlights

• Cinnamic acid improved growth performance and nutrient digestibility in lambs.
• Magnetized water enhanced antioxidant status and meat quality traits.
• Combined supplementation reduced lipid oxidation and improved meat color.
• Cinnamic acid shifted ruminal fermentation toward greater propionate production.
• The combination of cinnamic acid and 6000 Gauss water produced the best responses.

## Graphical abstract

**Figure:**
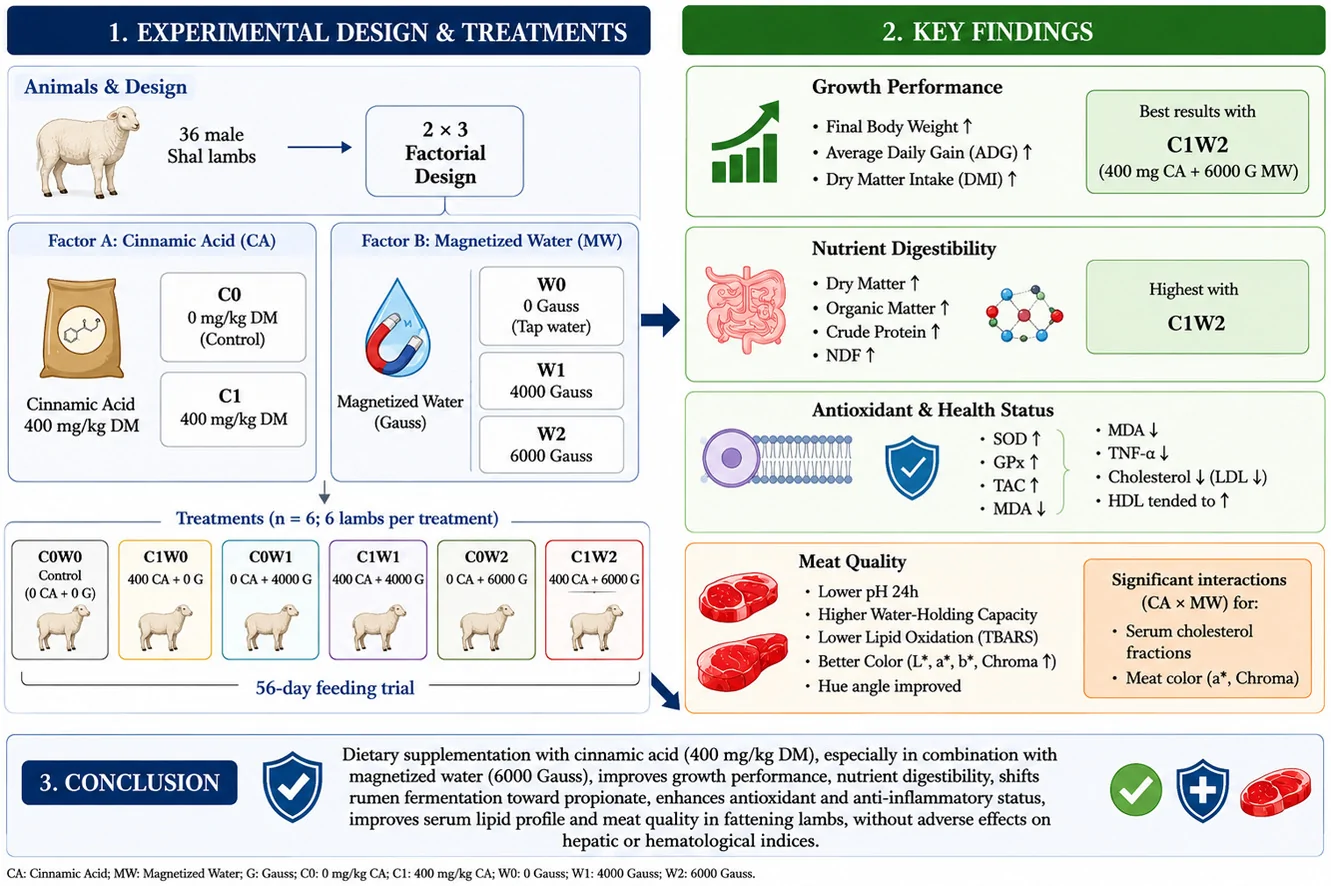

## 1 Introduction

The global demand for high-quality, safe, and affordable animal protein continues to rise, placing significant pressure on livestock production systems to enhance efficiency and product quality (Paim et al., 2019). In intensive lamb fattening systems, achieving optimal growth performance and feed efficiency is paramount. However, the high metabolic rates associated with rapid growth and development can increase the production of reactive oxygen species (ROS), leading to oxidative stress, which negatively impacts animal health, productivity, and meat quality (Liu et al., 2018; Orzuna-Orzuna et al., 2023). Consequently, there is growing research interest in identifying natural, safe, and effective dietary strategies to mitigate oxidative stress and improve overall performance (Valadez-García et al., 2021; Dorantes-Iturbide et al., 2022).

Plant-derived phenolic compounds, particularly hydroxycinnamic acids (HAs), represent one of the most promising classes of natural compounds. HAs are found abundantly in a wide variety of plant species, including fruits, vegetables, and grains (Peña-Torres et al., 2019; Sova and Saso, 2020). Cinnamic acid (CA) is a prominent member of this group, known for its potent antioxidant, anti-inflammatory, immunomodulatory, and antimicrobial properties (Yazdi et al., 2023; Song et al., 2013; Ogunlakin et al., 2024). In small ruminants, several studies have evaluated the physiological and productive impacts of HAs. Dietary supplementation with ferulic acid at 300 or 600 mg/d for 32 d in male hair lambs (Dorper × Pelibuey; 24 kg BW) led to enhanced final body weight (BW) and average daily gain (ADG), accompanied by oxidative muscle fiber hypertrophy and up-regulation of MHC I gene expression (Peña-Torres et al., 2022). Similarly, incorporating 300 mg/kg ferulic acid for 40 d into the diet of Katahdin lambs (33.7 kg) reduced feed intake without compromising body weight gain (BWG), while simultaneously improving meat color parameters such as L*, a*, and chroma (Tánori-Lozano et al., 2022). Under environmental stress, supplementing crossbred lambs exposed to cold conditions with 80 mg/kg FA for 30 d enhanced ADG, feed efficiency, and plasma antioxidant enzyme activities (glutathione peroxidase: GPx and catalase: CAT) alongside a reduction in malondialdehyde (MDA) levels; notably, a higher dosage of 2000 mg/kg exerted pro-oxidant effects, demonstrating a distinct dose-dependent action (Wang et al., 2019). Beyond purified ferulic acid, natural sources rich in cinnamic and ferulic acids, such as mangosteen peel powder (41.25 g/kg DM), have been shown to increase organic matter digestibility, total volatile fatty acids (VFA), and systemic antioxidant capacity, while effectively suppressing ruminal methane emissions in meat goats (Thai native × Anglo Nubian; 23.1 kg) over a 38-d period (Ban et al., 2022). Additionally, oral administration of an aqueous extract from *Caesalpinia coriaria* (30 or 60 mL/d for 60 d), which is characterized by high levels of ferulic and CA, dose-dependently improved ADG and feed intake while reducing fecal egg counts in growing lambs (18–20 kg) (Hernández Ruiz et al., 2024). Collectively, these findings demonstrate that HAs enhance growth performance, antioxidant status, and overall health in small ruminants, although the magnitude of the response depends on dosage, basal diet, species, and environmental conditions.

In parallel, the use of magnetized water (MW) has gained attention as a non-invasive, environmentally friendly, and low-cost technique to influence biological systems (Yacout et al., 2015). Exposing water to a magnetic field alters its physicochemical properties, including pH, surface tension, electrical conductivity (EC), and mineral solubility (Yacout et al., 2015; Ebrahim and Azab, 2017; Abdullaziz et al., 2022). These changes are believed to enhance water permeability across cell membranes, improving nutrient absorption, metabolic activity, and waste elimination (Cho and Lee, 2005; Al-Mufarrej et al., 2005; Lin and Yotvat, 1990). In small ruminants, several studies have evaluated the impacts of MW consumption across different physiological stages and production systems. In lactating Zaraibi goats (aged 2.5–3.5 years), drinking water treated with a magnetic field of 1200 or 3600 Gauss for 60 days significantly enhanced milk yield and composition (fat, protein, and lactose), with the greatest responses observed at 3600 Gauss; this was accompanied by improved ruminal fermentation through elevated total VFA concentrations and microbial protein synthesis, alongside decreased ammonia-N levels and enteric methane production (Yacout et al., 2015). Over a longer 6-month lactation period in Zaraibi goats (3–4 years; 32 kg), MW at 2000 or 4000 Gauss similarly increased daily and total milk yield, fat, protein, and solids-not-fat percentages, reaching maximum levels at 4000 Gauss (El-Kheshen et al., 2024). During late gestation and early lactation, Awassi ewes (2.5–3 years; 50–55 kg) receiving MW at 2000, 4000, or 6000 Gauss exhibited increased milk yield and improved milk composition, as well as higher serum total protein and high-density lipoprotein (HDL) levels, alongside reductions in cholesterol, LDL, and liver enzyme activities, with the 6000 Gauss treatment yielding the most pronounced benefits (Mustafa and Muhammed, 2023). In growing Baladi bucks (one year old; 25 kg), consumption of MW at 3600 Gauss for 60 days improved BWG and serum metabolites (total protein, albumin, globulin), while lowering aspartate aminotransferase (AST) and alanine aminotransferase (ALT), and blood urea nitrogen (BUN) levels; these metabolic changes coincided with lower ruminal pH, elevated microbial and protozoal counts, increased total antioxidant capacity (TAC), and reduced MDA concentrations (Abdullaziz et al., 2022). Furthermore, in weaned male small-tailed Han lambs (30 days old; 8 kg), long-term administration of MW over 240 days increased ADG by 27.4% and enhanced the apparent digestibility of dry matter, organic matter (OM), crude protein (CP), and energy (Cheng et al., 2016). Despite these promising individual effects, research on the potential synergistic or combined application of CA and MW in fattening lambs is non-existent. To our knowledge, no study to date has investigated this innovative nutritional strategy in fattening lambs. The main strength and primary contribution of this study lie in its novel, integrative evaluation of combining a bio-functional phenolic acid (CA) with physical water manipulation (MW at different field strengths). By bridging the gap between natural bioactive phytogenic additives and eco-friendly physical water treatment, this research addresses a critical knowledge gap in optimizing nutrient utilization, systemic antioxidant capacity, and meat quality traits through non-pharmacological means.

We hypothesized that the combined administration of CA and MW would synergistically improve the aforementioned parameters, providing a novel, natural, and effective nutritional strategy to enhance lamb productivity and produce high-quality, nutritionally superior meat.

Therefore, the present study was designed to evaluate the individual and interactive effects of dietary CA (400 mg/kg DM) and MW (at 4000 or 6000 Gauss) on growth performance, nutrient digestibility, ruminal fermentation parameters, blood biochemical and antioxidant status, and meat quality in fattening lambs. By systematically assessing these multifaceted parameters, this work aims to establish a scientific foundation for actionable, cost-effective, and sustainable strategies in commercial small ruminant production systems.

## 2 Materials and Methods

### 2.1 Experimental site and ethical approval

This study was conducted at an industrial sheep farm located in Kashmar, Khorasan-e Razavi, Iran. All experimental procedures involving animals were approved by the Institutional Animal Care and Use Committee of the University of Torbat-e Jam (Approval No. 2231). The animals were cared for according to the guidelines of the National Research Council (NRC, 2007) for the care and use of laboratory animals.

### 2.2 Animals and experimental design

A total of 36 intact male Shal lambs aged 4–5 months with an initial BW of 25.0 ± 2 kg (mean ± SD) were used in this study. The lambs were randomly assigned to six experimental groups (n = 6 per group) in a completely randomized design with a 2 × 3 factorial arrangement.

The sample size of six lambs per treatment was selected based on the availability of a homogeneous cohort of animals, the completely randomized factorial design, and animal welfare guidelines (NRC, 2007) to minimize animal use. Although an a priori statistical power analysis was not performed, which represents a statistical limitation, this sample size aligns with those typically used in small ruminant nutrition studies evaluating physiological and performance parameters (Ricci et al., 2020). However, the statistical implications of this sample size for detecting small-to-moderate treatment effects are discussed in the limitations section.

The factors consisted of two levels of CA (0 and 400 mg/kg dry matter: DM) and three levels of MW (0 Gauss, 4000 Gauss, and 6000 Gauss). The supplemental level of CA (400 mg/kg DM) was selected based on previous dose-response trials in small ruminants, where HAs levels between 300 and 600 mg/kg DM effectively optimized ruminal fermentation and antioxidant status without inducing pro-oxidant or adverse metabolic effects (Yang et al., 2010; Orzuna-Orzuna et al., 2023; Tian et al., 2023). The magnetic strength levels of 4000 and 6000 Gauss were chosen based on evidence demonstrating that exposure to this intensity range sufficiently alters water hydrogen-bonding networks, reduces surface tension, and enhances cellular membrane permeability, thereby maximizing nutrient solubility, microbial protein synthesis, and metabolic efficiency in ruminants without compromising physiological homeostasis (Mustafa and Muhammed, 2023; El-Kheshen et al., 2024).

The experimental treatments were as follows:

− **T1 (C0W0):** Control – basal diet + normal drinking water.
− **T2 (C0W1):** Basal diet + MW (4000 Gauss).
− **T3 (C0W2):** Basal diet + MW (6000 Gauss).
− **T4 (C1W0):** Basal diet + 400 mg/kg CA + normal drinking water.
− **T5 (C1W1):** Basal diet + 400 mg/kg CA + MW (4000 Gauss).
− **T6 (C1W2):** Basal diet + 400 mg/kg CA + MW (6000 Gauss).

All lambs were individually housed in metabolic cages (1.2 m × 0.8 m × 0.9 m) equipped with individual feeders and automatic waterers. The animals were acclimated to the experimental conditions and the basal diet for 14 days prior to the start of the trial. The experiment lasted for 56 days (8 weeks) of data collection following the adaptation period. A schematic overview of the experimental design and study procedures is shown in Fig. 1.

**Fig. 1:**
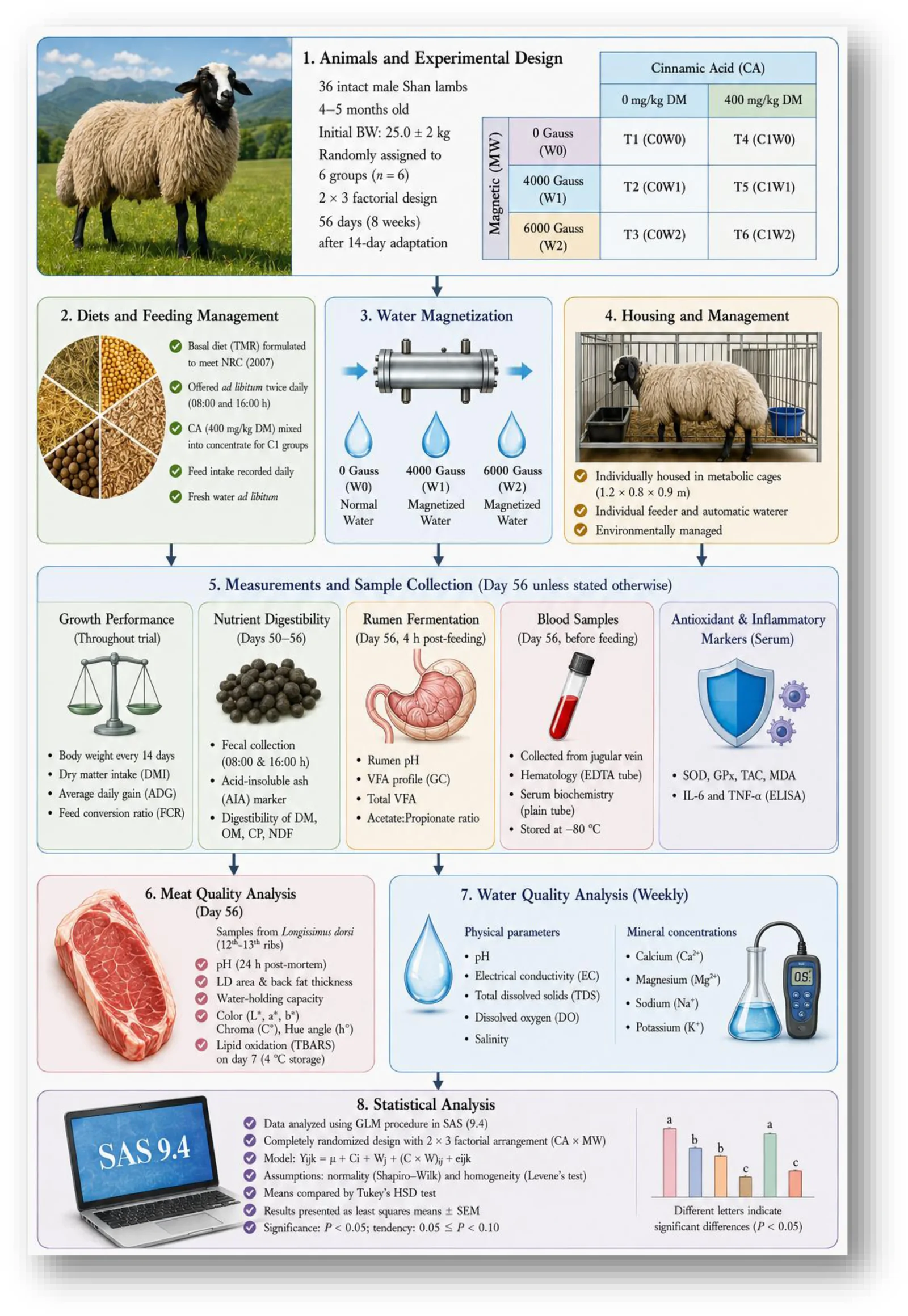
Schematic overview of the experimental design and study procedures.

### 2.3 Experimental diets and feeding management

The basal diet was formulated to meet or exceed the nutrient requirements of growing lambs according to the National Research Council (NRC, 2007) recommendations. The diet was provided as a total mixed ration (TMR) and consisted of forage and concentrate ingredients. The ingredient composition and chemical analysis of the basal diet are presented in Table 1.

**Table 1:** Ingredients and chemical composition of the basal diet (DM basis).

Ingredients	Amount (% of diet DM)
Alfalfa hay	18.0
Wheat straw	7.0
Ground corn	32.0
Ground barley	24.0
Wheat bran	10.0
Soybean meal	7.0
Vitamin-mineral supplement^1^	1.0
Magnesium oxide	0.5
Salt	0.5
**Total**	**100.0**
	

**Chemical composition**	

Dry matter (% of as fed)	89.6
Crude protein (%)	15.7
Neutral detergent fiber (%)	33.8
Acid detergent fiber (%)	17.9
Ether extract (%)	2.9
Non-fiber carbohydrates (%)	41.4
Metabolizable energy (Mcal/kg DM)	2.63
Ash (%)	6.2
Calcium (%)	0.72
Phosphorus (%)	0.39

DM: dry matter.

1 The vitamin-mineral supplement provided the following per kilogram of diet DM: 10,000 IU of vitamin A, 2,000 IU of vitamin D_3_, 25 mg of vitamin E, 40 mg of Zn (as ZnSO_4_), 40 mg of Mn (as MnSO_4_), 30 mg of Fe (as FeSO_4_), 10 mg of Cu (as CuSO_4_), 0.5 mg of I (as KI), 0.1 mg of Co (as CoCl_2_), and 0.3 mg of Se (as Na_2_SeO_3_).Metabolizable energy values were calculated from the analyzed chemical composition according to NRC (2007) prediction equations and were not determined experimentally.

All diets were offered ad libitum twice daily at 08:00 and 16:00 h. Feed offered and orts were recorded daily to calculate dry matter intake (DMI). Fresh, clean drinking water was available ad libitum throughout the experiment. For groups receiving MW (W1 and W2), water was treated using a commercial magnetic water conditioning system (Baotou INST Magnetic New Materials Co., Baotou, China) with magnetic field intensities of 4000 Gauss and 6000 Gauss, respectively. The MW was prepared fresh every 12 h and offered to the animals in separate waterers.

### 2.4 Cinnamic acid supplementation

Trans-cinnamic acid (purity ≥ 99%, CAS No. 140-10-3; Sigma-Aldrich, St. Louis, MO, USA) was incorporated into the concentrate portion of the diet at a dosage of 400 mg/kg of DM. To ensure uniform distribution, the CA was initially pre-mixed with finely ground corn as a carrier and then gradually integrated into the entire concentrate mixture. The supplemented concentrate was subsequently mixed with the forage components to prepare the TMR. The stability of CA within the feed matrix was verified via HPLC analysis, following the protocol described by Kopjar et al. (2021).

### 2.5 Water magnetization

The MW used in this study was produced from the farm's tap water supply using a permanent magnetic water conditioning system (Baotou INST Magnetic New Materials Co., Baotou, China). The system consisted of permanent magnets generating magnetic fields of 4000 Gauss (W1) and 6000 Gauss (W2). The strength of the magnets was verified using a digital Gauss meter (Model GM-2; AlphaLab Inc., Salt Lake City, UT, USA) before the initiation and after the termination of the experiment. The water was passed through the magnetic field in a single pass at a flow rate of 2 L/min, without recirculation, before being offered to the animals. The treated water was used within 60 minutes of treatment to maintain its properties, following the recommendations of Lin and Yotvat (1990).

### 2.6 Water quality analysis

Samples of normal water and MW were collected weekly and analyzed for physicochemical properties according to standard methods. Physical parameters including pH, EC, total dissolved solids (TDS), and salinity were measured using a portable multi-parameter meter (HI98194; Hanna Instruments, Woonsocket, RI, USA). Dissolved oxygen (DO) was measured using a DO meter (Oxi 3310; WTW, Weilheim, Germany). Mineral concentrations (Ca^2+^, Mg^2+^, Na^+^, K^+^) were determined using atomic absorption spectrophotometry (AA-7000; Shimadzu Corporation, Kyoto, Japan) following the method of Hegedűs-Mîndru et al. (2013). The detailed physicochemical properties of normal water and MW are presented in Table 2.

**Table 2:** Physicochemical properties of normal and magnetized water used in the experiment.

	Unit	Normal Water (0 Gauss)	Magnetized Water (4000 Gauss)	Magnetized Water (6000 Gauss)
**Physical parameters**				
pH	-	7.12	7.46	7.71
Electrical conductivity	µS/cm	450	468	481
Total dissolved solids	mg/L	225	234	241
Dissolved oxygen	mg/L	6.8	7.2	7.5
Salinity	ppm	0.22	0.23	0.24
				

**Mineral concentrations**				

Calcium (Ca²⁺)	mg/L	42.5	42.7	42.6
Magnesium (Mg²⁺)	mg/L	18.3	18.4	18.5
Sodium (Na⁺)	mg/L	28.4	28.6	28.5
Potassium (K⁺)	mg/L	3.2	3.2	3.3

Data are presented as mean values of triplicate measurements from composite water samples to describe initial water parameters; no formal statistical testing was performed.

### 2.7 Growth performance parameters

The BW of individual lambs was recorded at the beginning of the experiment (day 0), then every 14 days, and at the end of the experiment (day 56) using a digital scale (± 0.1 kg). The BWG was calculated as the difference between final and initial BW. The ADG was calculated by dividing total BWG by the number of experimental days.

Feed intake was recorded daily by weighing the amount of feed offered and the orts remaining. DMI was calculated based on the DM content of the feed and orts. Feed conversion ratio (FCR) was calculated as DMI divided by ADG.

### 2.8 Nutrient digestibility

Apparent nutrient digestibility was determined using the acid-insoluble ash (AIA) method as an internal indigestible marker according to the procedure described by Sein and Todd (1988). Fecal grab samples were collected directly from the rectum of each lamb twice daily (08:00 and 16:00 h) during the last 7 days of the experiment (days 50–56). The collected fecal samples were pooled per animal, dried in a forced-air oven at 60°C for 48 h, ground to pass through a 1-mm screen, and stored in airtight containers for subsequent analysis.

The AIA content of feed and fecal samples was determined according to the method of Van Keulen and Young (1977). Briefly, 2 g of dried sample was ashed in a muffle furnace at 550°C for 8 h. The ash was then boiled with 100 mL of 2N HCl for 5 min and filtered through ashless filter paper. The residue was washed with distilled water, dried, and re-ashed at 550°C for 2 h. The AIA content was calculated as the weight of the residue after acid treatment.

The apparent digestibility coefficients for DM, OM, CP, and neutral detergent fiber (NDF) were calculated using the following formula:

Digestibility (%) = 100 – [100 × (AIA in feed / AIA in feces) × (Nutrient in feces / Nutrient in feed)]

Feed and fecal samples were analyzed for chemical composition according to the methods of the Association of Official Analytical Chemists (AOAC, 2005). The DM was determined by oven drying at 100°C for 24 h (Method 934.01). The CP was calculated as N × 6.25 using the Kjeldahl method (Method 984.13). Ether extract was determined by petroleum ether extraction using a Soxtec system (Method 920.39). Ash was determined by incineration in a muffle furnace at 600°C for 4 h (Method 942.05). The NDF and acid detergent fiber (ADF) were determined using an ANKOM 200 Fiber Analyzer (ANKOM Technology, Macedon, NY, USA) following the procedure of Van Soest et al. (1991).

### 2.9 Ruminal fermentation parameters

Rumen fluid samples were collected from each lamb at the end of the experiment (day 56) at 4 h post-feeding using a stomach tube fitted with a suction pump. The first few milliliters were discarded to minimize contamination with saliva. The collected rumen fluid (approximately 100 mL) was strained through four layers of cheesecloth into sterile plastic containers and immediately transported to the laboratory for analysis (Kazemi, 2024).

The pH of the rumen fluid was measured immediately upon collection using a digital pH meter (pH 700; Eutech Instruments, Singapore) according to the standard method described by Nordlund and Garrett (1994). For VFA analysis, 10 mL of strained rumen fluid was mixed with 2 mL of metaphosphoric acid (25% w/v) and centrifuged at 10,000 × g for 15 minutes at 4°C (Sorvall Legend XT/XF Centrifuge Series, Thermo Fisher Scientific, Waltham, MA, USA). The supernatant was stored at –20°C until analysis. VFA concentrations, including acetate, propionate, butyrate, isobutyrate, valerate, and isovalerate, were determined using gas chromatography (Agilent 6890 GC, Agilent Technologies, Wilmington, DE, USA) equipped with a flame ionization detector (FID) and a 30 m × 0.25 mm × 0.25 µm DB-FFAP capillary column, following the procedure described by Erwin et al. (1961) with modifications. The total VFA concentration was calculated as the sum of individual VFA, and the acetate-to-propionate ratio (A:P) was determined by dividing acetate concentration by propionate concentration.

### 2.10 Blood sample collection and analysis

On day 56 of the experiment, blood samples (approximately 10 mL per lamb) were collected from the jugular vein using Vacutainer tubes (Becton Dickinson, Franklin Lakes, NJ, USA) before the morning feeding (Smith, 2020). The blood was distributed into two types of tubes: tubes containing EDTA (K3EDTA, 1.5 mg/mL) for hematological analysis, and plain tubes (without anticoagulant) for serum separation.

For serum separation, blood samples in plain tubes were allowed to clot for 30 minutes at room temperature and then centrifuged at 3,000 × *g* for 15 minutes at 4°C. The resulting serum was aliquoted into Eppendorf tubes and stored at –80°C until biochemical analysis.

### 2.11 Hematological parameters

Hematological parameters, including RBC count, white blood cell (WBC) count, hemoglobin (Hb) concentration, and hematocrit (Hct), were measured immediately after blood collection using an automated veterinary hematology analyzer (Exigo EOS; Boule Medical AB, Stockholm, Sweden) according to the manufacturer's instructions and standard veterinary clinical procedures (Thrall et al., 2012; Ortín et al., 2026).

### 2.12 Serum biochemical parameters

Serum concentrations of glucose, BUN, total cholesterol, HDL, low-density lipoprotein (LDL), triglycerides (TG), as well as AST and ALT activities were measured using commercial colorimetric assay kits (Pars Azmoon Co., Tehran, Iran) according to the manufacturer's protocols, following standard veterinary clinical chemistry procedures (Kaneko et al., 2008). These commercial diagnostic kits have been previously validated and extensively applied for lipid profiling and serum biochemical determinations in ovine studies (Azimzadeh and Javadi, 2020; Omidi and Nik, 2024). All analyses were performed using an automated biochemistry analyzer (BT 3000 Plus, Biotecnica Instruments S.p.A., Rome, Italy).

### 2.13 Antioxidant status and inflammatory markers

#### 2.13.1 Superoxide dismutase activity

The superoxide dismutase (SOD) activity was determined using a commercial colorimetric assay kit (Bioassay Technology Laboratory, Shanghai, China), following the manufacturer’s instructions based on the tetrazolium salt inhibition assay, adapted from the principle described by Beauchamp and Fridovich (1971). Absorbance was measured at 450 nm using a microplate reader (Synergy H1 Hybrid Reader, BioTek Instruments Inc., Winooski, VT, USA). SOD activity was expressed as U/mL.

#### 2.13.2 Glutathione peroxidase activity

Serum GPx activity was determined using a commercial colorimetric assay kit (GPx Assay Kit, Cayman Chemical Co., Ann Arbor, MI, USA) according to the method described by Paglia and Valentine (1967). The assay measures GPx activity indirectly through a coupled reaction with glutathione reductase, in which GPx catalyzes the reduction of cumene hydroperoxide while oxidizing reduced glutathione (GSH). Oxidized glutathione is subsequently converted back to GSH by glutathione reductase in the presence of NADPH. The oxidation of NADPH to NADP+ was monitored by measuring the decrease in absorbance at 340 nm using a microplate reader. One unit of GPx activity was defined as the amount of enzyme catalyzing the oxidation of NADPH per minute, and the results were expressed as U/L.

#### 2.13.3 Total antioxidant capacity

Serum TAC was determined using a commercial colorimetric assay kit (TAC Assay Kit, BioVision Inc., Milpitas, CA, USA) according to the manufacturer's instructions. The assay is based on the reduction of Cu²⁺ to Cu⁺ by antioxidants present in the sample, as described for the CUPRAC method by Apak et al. (2007). The reduced Cu⁺ subsequently forms a colored complex with a chromogenic reagent, and the absorbance was measured at 570 nm using a microplate reader. TAC values were calculated from a standard curve generated using Trolox (6-hydroxy-2,5,7,8-tetramethylchroman-2-carboxylic acid) as the reference antioxidant and expressed as mmol Trolox equivalents/L.

#### 2.13.4 Malondialdehyde

Serum MDA concentration, an indicator of lipid peroxidation, was determined using the thiobarbituric acid reactive substances (TBARS) assay according to the method of Ohkawa et al. (1979). Briefly, 0.2 mL of serum was mixed with 0.2 mL of 8.1% sodium dodecyl sulfate (SDS), 1.5 mL of 20% acetic acid (pH 3.5), and 1.5 mL of 0.8% thiobarbituric acid (TBA). The mixture was heated in a boiling water bath for 60 min and subsequently cooled to room temperature. Then, 2.5 mL of butanol–pyridine solution (15:1, v/v) was added, followed by centrifugation at 4,000 × *g* for 10 min. The absorbance of the upper organic phase was measured at 532 nm using a spectrophotometer. MDA concentration was calculated using a standard curve prepared from 1,1,3,3-tetraethoxypropane and expressed as nmol/mL.

#### 2.13.5 Interleukin-6 and tumor necrosis factor-alpha

Serum concentrations of interleukin-6 (IL-6) and tumor necrosis factor-α (TNF-α) were determined using commercial sandwich ELISA kits (Invitrogen, Carlsbad, CA, USA) according to the manufacturer's instructions, as previously described for ovine cytokine quantification (Bouquet et al., 2020).

### 2.14 Meat quality analysis

At the end of the experiment (day 56), all lambs were fasted for 12 h with free access to water. The animals were transported to a commercial abattoir and humanely slaughtered according to standard halal procedures and the guidelines for animal welfare. After exsanguination, the carcasses were skinned, eviscerated, and weighed to determine hot carcass weight. Hot dressing percentage was calculated as (hot carcass weight / final live weight) × 100. The carcasses were then chilled at 4°C for 24 h. Samples were taken from the *longissimus dorsi* (LD) muscle between the 12^th^ and 13^th^ ribs for subsequent analysis of pH, color, and chemical composition, according to the guidelines of the American Meat Science Association (AMSA, 2012).

#### 2.14.1 pH measurement

The pH of the LD muscle (between the 12^th^ and 13^th^ ribs) was measured at 24 h post-mortem using a portable digital pH meter (Testo 205; Testo SE & Co. KGaA, Lenzkirch, Germany) equipped with a puncture-type glass electrode. The pH meter was calibrated with standard buffer solutions of pH 4.0 and 7.0 prior to each measurement (AMSA, 2012). Three measurements were taken per sample, and the mean value was recorded.

#### 2.14.2 *Longissimus dorsi* area and back fat thickness

The LD muscle area was measured from the cross-section at the level of the 12^th^–13^th^ ribs. The outline of the LD muscle was traced on tracing paper, and the area was determined using a digital planimeter (Placom KP-90N; Koizumi Trading Co., Ltd., Tokyo, Japan) as outlined by AMSA (2012). Results were expressed in square centimeters (cm²).

Back fat thickness was measured at the same anatomical location (between the 12^th^ and 13^th^ ribs) using a digital caliper according to standard carcass grading guidelines (USDA, 1992).

#### 2.14.3 Water-holding capacity

Water‑holding capacity (WHC) was determined by the filter paper press method, as described by Kang et al. (2022).

#### 2.14.4 Color measurements

Color parameters (CIE L*, a*, b*) of the LD muscle were measured at 24 h post-mortem on the freshly cut surface after 30 min of blooming at 4°C using a portable colorimeter (Color Tec PCM+; Color Tec Associates Inc., Clinton, NJ, USA) according to the AMSA Meat Color Measurement Guidelines (AMSA, 2012). Prior to measurements, the colorimeter was calibrated using a standard white tile provided by the manufacturer. Three measurements were taken at different locations on each sample, and the average values were used for statistical analysis. Additional color indices were calculated using the following formulas (AMSA, 2012):

− Chroma (color saturation, C*) = √[(a*)² + (b*)²]
− Hue angle (*h°*) = atan2(b*, a*) × (180/*π*) (expressed in degrees)

#### 2.14.5 Lipid oxidation (TBARS)

Lipid oxidation in the LD muscle was assessed by measuring TBARS according to Buege and Aust (1978), with minor modifications. Meat samples (5 g) were stored at 4°C for 7 days to simulate retail display conditions, and TBARS was determined on day 7.

Briefly, 5 g of minced meat sample was homogenized with 15 mL of 10% (w/v) trichloroacetic acid (TCA) containing 0.01% (w/v) butylated hydroxytoluene (BHT). The homogenate was filtered through Whatman No. 1 filter paper (or centrifuged, if applicable).

Subsequently, 2 mL of the filtrate was mixed with 2 mL of 0.02 M TBA solution and incubated in a boiling water bath for 30 min. After cooling, absorbance was measured at 532 nm using a spectrophotometer.

TBARS values were calculated from a standard curve prepared using 1,1,3,3-tetraethoxypropane and expressed as mg MDA per kg of meat (mg MDA/kg).

### 2.15 Statistical analysis

All data were subjected to statistical analysis using the General Linear Model (GLM) procedure of SAS software (version 9.4; SAS Institute Inc., Cary, NC, USA). The data were analyzed as a completely randomized design with a 2 × 3 factorial arrangement of treatments (two levels of CA × three levels of MW). The statistical model used was:

*Y_ijk_* = *μ* + C*_i_* + W*_j_* + (C × W)*_ij_* + *ε_ijk_*

where:

• *Y_ijk_* = the observed value
• *μ* = the overall mean
• C*_i_* = the fixed effect of CA (*i* = 0, 400 mg/kg)
• W*_j_* = the fixed effect of MW (*j* = 0, 4000, 6000 Gauss)
• (C × W)*_ij_* = the interaction effect between CA and MW
• *ε_ijk_* = the random error term

Prior to analysis, all data were tested for normality and homogeneity of variances using the Shapiro-Wilk test and Levene's test, respectively. Treatment means were compared using Tukey's honestly significant difference (HSD) post hoc test when significant differences were detected (*P* < 0.05). The results are presented as least squares means with pooled standard error of the mean (SEM). Significance was declared at *P* < 0.05, and tendencies were considered at 0.05 ≤ *P* < 0.10.

Given the sample size (n = 6 per treatment) and the absence of an a priori power calculation, statistical power to detect small-to-moderate main effects or two-way interactions (C × W) may be restricted. Therefore, non-significant statistical findings (*P* > 0.05) were interpreted with caution and not definitively regarded as an absence of a biological effect.

## 3 Results

Magnetization of drinking water at 4000 and 6000 Gauss resulted in numerical changes in several physicochemical properties of water (Table 2). Compared with normal water, MW at 6000 Gauss exhibited higher pH (7.71 vs. 7.12), EC (481 vs. 450 µS/cm), and DO concentration (7.5 vs. 6.8 mg/L). Minor increases were also observed in TDS and salinity, whereas mineral concentrations remained relatively unchanged among treatments.

### 3.1 Growth performance and feed intake

The effects of CA and MW on growth performance and feed intake are presented in Table 3. Dietary CA supplementation increased final BW (*P* < 0.01), ADG (*P* = 0.02), and DMI (*P* = 0.03) compared with the control treatments. Lambs receiving CA exhibited greater growth performance than those fed the unsupplemented diets. Final BW ranged from 37.8 to 40.5 kg, whereas ADG ranged from 236 to 270 g/day across treatments. MW had no significant effect on final BW, ADG, DMI, or FCR (*P* > 0.05), although numerically greater values for BW and ADG were observed at 6000 Gauss. Neither CA nor MW treatments affected the FCR (*P* > 0.05). In addition, no significant CA × MW interactions were detected for any growth performance variables (*P* > 0.05).

**Table 3:** Effects of cinnamic acid and magnetized water on growth performance, nutrient digestibility, and ruminal fermentation parameters in lambs.

								*P*-value		
**Trait**	C0W0	C0W1	C0W2	C1W0	C1W1	C1W2	SEM	C	W	C×W
DMI (kg/d)	1.49	1.53	1.52	1.55	1.59	1.57	0.03	0.03	0.40	0.98
Final BW (kg)	37.8	38.4	38.9	39.1	39.7	40.5	0.57	< 0.01	0.10	0.94
ADG (g/day)	236	240	256	255	266	270	10.0	0.02	0.23	0.80
FCR	6.36	6.42	6.01	6.15	5.99	5.85	0.26	0.22	0.42	0.87
DMD (%)	67.7	68.6	69.9	70.7	71.4	72.7	0.74	< 0.001	0.03	0.99
OMD (%)	69.0	69.7	71.2	72.0	73.0	74.3	0.57	< 0.001	0.02	0.95
CPD (%)	70.7	71.2	73.2	71.5	74.1	75.4	0.60	< 0.001	< 0.001	0.19
NDFD (%)	48.6	49.1	50.4	50.2	52.7	53.2	0.50	< 0.001	< 0.001	0.16
										

**Ruminal VFA**										

Total VFA	62.8	63.7	62.2	61.1	60.6	60.3	0.58	< 0.001	0.24	0.43
Acetate	63.0	63.4	62.9	61.5	61.0	60.1	0.54	< 0.001	0.31	0.47
Propionate	19.1	19.9	20.6	20.9	21.6	22.3	0.37	< 0.001	< 0.01	0.97
Butyrate	13.2	13.4	13.5	13.0	13.1	13.3	0.20	0.15	0.35	0.91
Isobutyrate	1.18	1.10	1.11	1.09	1.18	1.11	0.07	0.93	0.89	0.44
Valerate	0.74	0.68	0.77	0.71	0.82	0.88	0.04	0.02	0.03	0.07
Isovalerate	1.05	1.20	1.08	1.15	1.20	1.16	0.06	0.19	0.23	0.64
A:P	3.30	3.19	3.06	2.94	2.83	2.71	0.06	< 0.001	< 0.01	0.99

DMI, dry matter intake; BW, body weight; ADG, average daily gain; FCR, feed conversion ratio; DMD, dry matter digestibility; OMD, organic matter digestibility; CPD, crude protein digestibility; NDFD, neutral detergent fiber digestibility. VFA, volatile fatty acids; A:P, acetate: propionate ratio.

All VFA components except total VFA are expressed in mol/100 mol. Total VFA unit is mmol/L.

C0 = 0 mg/kg DM cinnamic acid, C1 = 400 mg/kg DM; W0 = 0 Gauss, W1 = 4000 Gauss, W2 = 6000 Gauss.

SEM, standard error of the mean.

### 3.2 Nutrient digestibility

Dry matter digestibility (DMD) and organic matter digestibility (OMD) were significantly increased by CA supplementation (*P* < 0.001) and MW treatment (*P* < 0.05; Table 3). Lambs receiving CA combined with 6000 Gauss MW (C1W2) showed the highest DMD and OMD values (*P* < 0.001). Crude protein digestibility (CPD) and neutral detergent fiber digestibility (NDFD) were also significantly improved by both CA and MW treatments (*P* < 0.001). Lambs in the C1W2 treatment showed the greatest CPD (75.4%) and NDFD (53.2%) values. However, no significant CA × MW interactions were detected for DMD, OMD, CPD, or NDFD (*P* > 0.05).

### 3.3 Ruminal fermentation parameters

Ruminal fermentation parameters are presented in Table 3. Total VFA concentration was significantly reduced by CA supplementation (*P* < 0.001), with lower values observed in CA-treated groups than in control groups. Acetate proportion was significantly decreased by CA (*P* < 0.001), whereas propionate proportion was significantly increased by both CA (*P* < 0.001) and MW (*P* < 0.01). Consequently, the acetate-to-propionate (A:P) ratio was significantly reduced by CA (*P* < 0.001) and MW (*P* < 0.01), with the lowest ratio observed in the C1W2 group (2.71). Valerate concentration was significantly affected by both CA (*P* = 0.02) and MW (*P* = 0.03), and a tendency for CA × MW interaction was observed (*P* = 0.07). However, butyrate, isobutyrate, and isovalerate concentrations were not significantly influenced by treatments (*P* > 0.05). No significant CA × MW interactions were detected for the remaining ruminal fermentation parameters (*P* > 0.05).

### 3.4 Blood biochemical parameters

The effects of CA and MW on blood biochemical parameters are shown in Table 4. Glucose concentration and hematological parameters including Hb, Hct, RBC, WBC, and BUN were not significantly affected by treatments (*P* > 0.05).

**Table 4:** Effects of cinnamic acid and magnetic water on blood metabolites, antioxidant status, and inflammatory markers in lambs.

								*P*-value		
Trait	C0W0	C0W1	C0W2	C1W0	C1W1	C1W2	SEM	C	W	C×W
Glucose (mg/dL)	63.4	63.2	62.2	60.8	62.8	63.1	1.04	0.43	0.68	0.25
Hb (g/dL)	10.5	10.8	10.8	10.7	10.8	10.7	0.22	0.82	0.51	0.76
Hct (%)	35.4	35.1	35.6	35.5	35.1	34.8	0.54	0.68	0.81	0.63
BUN (mg/dL)	18.9	19.0	19.6	19.6	19.0	19.2	0.45	0.75	0.63	0.52
RBC (×10¹²/L)	10.8	10.9	10.6	10.9	10.4	10.7	0.18	0.52	0.48	0.32
WBC (×10^9^/L)	8.47	8.57	8.45	8.67	8.83	8.53	0.19	0.25	0.55	0.89
Cholesterol (mg/dL)	74.2^a^	73.4^a^	73.7^a^	69.2^b^	73.6^a^	73.1^a^	0.48	< 0.001	< 0.01	< 0.001
HDL (mg/dL)	35.1^b^	35.9^b^	35.1^b^	38.5^a^	34.8^b^	34.9^b^	0.43	0.06	< 0.001	< 0.001
LDL (mg/dL)	32.2^a^	30.7^a^	31.8^a^	24.2^b^	32.0^a^	31.6^a^	0.75	< 0.001	< 0.01	< 0.001
Triglyceride (mg/dL)	34.6	34.1	34.2	32.9	34.2	33.2	0.54	0.06	0.70	0.23
AST (U/L)	85.5	86.4	85.3	86.1	86.4	85.5	0.75	0.67	0.43	0.93
ALT (U/L)	24.2	23.6	24.1	23.8	24.7	24.9	0.45	0.20	0.60	0.23
SOD (U/mL)	4.17	4.67	5.13	5.30	5.50	5.45	0.20	< 0.001	0.03	0.13
GPx (U/L)	182	184	188	193	197	201	2.67	< 0.001	0.06	0.92
TAC (mmol/L)	1.06	1.09	1.09	1.22	1.32	1.44	0.05	< 0.001	0.04	0.14
MDA (nmol/mL)	2.18	2.02	2.00	1.99	1.82	1.69	0.07	< 0.001	< 0.01	0.63
IL-6 (pg/mL)	18.3	18.0	18.2	17.8	17.4	16.3	0.77	0.12	0.59	0.59
TNF-α (pg/mL)	11.3	11.0	11.4	10.3	9.83	8.58	0.55	< 0.001	0.32	0.22

^a,b^ Means within a row with different superscripts differ significantly (*P* < 0.05) based on Tukey’s HSD test for significant interaction effects (C×W). For variables without significant interaction (*P* > 0.05), superscripts are omitted, and differences are discussed based on main effects (C and W). Hb, hemoglobin; Hct, hematocrit; BUN, blood urea nitrogen; RBC, red blood cells; WBC, white blood cells; AST, aspartate aminotransferase; ALT, alanine aminotransferase; SOD, superoxide dismutase; GPx, glutathione peroxidase; TAC, total antioxidant capacity; MDA, malondialdehyde; IL-6, interleukin 6; TNF-α, tumor necrosis factor alpha. C0 = 0 mg/kg DM cinnamic acid, C1 = 400 mg/kg DM; W0 = 0 Gauss, W1 = 4000 Gauss, W2 = 6000 Gauss. SEM, standard error of the mean.

Serum cholesterol concentration was significantly reduced by CA supplementation (*P* < 0.001), while MW also exerted a significant effect (*P* < 0.01). A significant CA × MW interaction was detected for cholesterol concentration (*P* < 0.001), with the lowest value observed in the C1W0 group (69.2 mg/dL). HDL concentration tended to increase with CA supplementation (*P* = 0.06), whereas MW significantly affected HDL levels (*P* < 0.001). A significant CA × MW interaction was also observed for HDL (*P* < 0.001). Similarly, LDL concentration was significantly reduced by CA (*P* < 0.001) and MW (*P* < 0.01), with a significant interaction between factors (*P* < 0.001). Triglyceride concentration was not significantly influenced by treatments (*P* > 0.05).

Activities of liver enzymes AST and ALT were not significantly altered by either CA or MW supplementation (*P* > 0.05), indicating no adverse effects of treatments on liver function.

### 3.5 Antioxidant status

CA supplementation significantly increased SOD activity (*P* < 0.001), while MW also significantly enhanced SOD activity (*P* = 0.03). GPx activity was significantly increased by CA supplementation (*P* < 0.001), while a tendency for increased GPx activity was observed with MW supplementation (*P* = 0.06). TAC was significantly improved by both CA (*P* < 0.001) and MW (*P* = 0.04), with the highest value observed in the C1W2 group (1.44 mmol/L). In contrast, MDA concentration was significantly reduced by CA (*P* < 0.001) and MW (*P* < 0.01), with the lowest value recorded in the C1W2 group (1.69 nmol/mL). No significant CA × MW interactions were observed for antioxidant-related variables (*P* > 0.05).

### 3.6 Inflammatory cytokines

IL-6 concentration was not significantly affected by CA or MW supplementation (*P* > 0.05), although numerically lower values were observed in the C1W2 group. TNF-α concentration was significantly reduced by CA supplementation (*P* < 0.001), whereas the effect of MW and the CA × MW interaction were not significant (*P* > 0.05). The lowest TNF-α concentration was observed in the C1W2 group (8.58 pg/mL).

### 3.7 Meat quality parameters

The effects of CA and MW on carcass traits and meat quality parameters are presented in Table 5. Meat pH measured at 24 h postmortem was significantly reduced by both CA and MW supplementation (*P* < 0.001), with the lowest value observed in the C1W2 group (5.69). Hot dressing percentage was significantly increased by CA supplementation (*P* = 0.04), whereas MW had no significant effect (*P* > 0.05). The LD area was significantly increased by CA supplementation (*P* < 0.001), and a significant CA × MW interaction was observed (*P* = 0.05), with the largest LD area recorded in the C1W2 group (12.9 cm²). Back fat thickness was not affected by treatments (*P* > 0.05).

**Table 5:** Effects of cinnamic acid and magnetized water on carcass traits and meat quality parameters in lambs.

								*P*-value		
**Trait**	C0W0	C0W1	C0W2	C1W0	C1W1	C1W2	SEM	C	W	C×W
pH (24 h postmortem)	5.81	5.76	5.76	5.75	5.72	5.69	0.01	< 0.001	< 0.001	0.47
Hot dressing percentage (%)	45.2	46.8	47.1	47.4	48.4	49.0	0.89	0.04	0.36	0.94
*Longissimus dorsi* area (cm²)	11.6^bc^	11.3^c^	11.6^bc^	11.7^bc^	12.6^ab^	12.9^a^	0.27	< 0.001	0.07	0.05
Back fat thickness (mm)	3.15	3.06	2.98	2.92	3.14	3.20	0.13	0.83	0.85	0.22
WHC (%)	61.7	62.2	65.6	68.2	71.6	72.0	0.66	< 0.001	< 0.001	0.06
TBARS day 7 (mg MDA/kg)	1.93	1.74	1.58	1.29	1.24	1.01	0.05	< 0.001	< 0.001	0.35
L*	39.8	38.1	38.8	40.3	41.0	41.2	0.51	< 0.001	0.55	0.06
a*	18.8bcd	17.8^d^	18.3^cd^	19.7^bc^	20.1^ab^	21.7^a^	0.41	< 0.001	0.06	0.02
b*	6.60	7.03	6.92	7.60	8.32	8.68	0.20	< 0.001	< 0.01	0.18
Chroma	19.9^bc^	19.2^d^	19.6^cd^	21.2^bc^	21.8^ab^	23.3^a^	0.39	< 0.001	0.03	0.01
Hue angle (°)	19.4	21.5	20.7	21.1	22.5	21.8	0.64	0.02	0.03	0.83

^a–d^ Means within a row with different lowercase superscripts differ significantly (*P* < 0.05) based on Tukey’s HSD test for traits with significant interaction effects (C × W). For traits without significant interaction (P > 0.05), superscripts are omitted, and results are evaluated based on main effects (C or W). WHC, water-holding capacity; TBARS, thiobarbituric acid reactive substances; L*, lightness; a*, redness; b*, yellowness; Chroma, color intensity; Hue angle. C0 = 0 mg/kg DM cinnamic acid, C1 = 400 mg/kg DM; W0 = 0 Gauss, W1 = 4000 Gauss, W2 = 6000 Gauss. SEM, standard error of the mean.

The WHC was significantly improved by both CA and MW supplementation (*P* < 0.001), with a tendency for CA × MW interaction (*P* = 0.06). Lipid oxidation, assessed as TBARS after 7 days of refrigerated storage, was significantly reduced by both CA and MW (*P* < 0.001), with the lowest value observed in the C1W2 group (1.01 mg MDA/kg).

Regarding meat color characteristics, lightness (L*) was significantly increased by CA supplementation (*P* < 0.001), while the CA × MW interaction tended to be significant (*P* = 0.06). Redness (a*) was significantly increased by CA (*P* < 0.001), with a significant CA × MW interaction (*P* = 0.02). Yellowness (b*) was significantly increased by both CA (*P* < 0.001) and MW (*P* < 0.01). Chroma was significantly affected by CA (*P* < 0.001), MW (*P* = 0.03), and their interaction (*P* = 0.01). Hue angle was significantly influenced by both CA (*P* = 0.02) and MW (*P* = 0.03).

Overall, the combined supplementation of CA and MW, particularly at 6000 Gauss, generally resulted in improved meat quality characteristics, including lower pH, greater WHC, reduced lipid oxidation, and improved color stability.

## 4 Discussion

### 4.1 Water quality and its physiological implications

Characterization of the drinking water revealed minor variations in its baseline physicochemical properties following magnetic exposure. As shown in Table 2, water exposed to a 6000 Gauss magnetic field exhibited values for pH (7.71 vs. 7.12), EC (481 vs. 450 µS/cm), and DO (7.5 vs. 6.8 mg/L) compared with untreated control water. In contrast, concentrations of Ca^2+^, Mg^2+^, Na^+^, and K^+^ showed minimal differences among water treatments. These shifts align with descriptive patterns reported by Yacout et al. (2015) and Abdullaziz et al. (2022), who observed altered pH, conductivity, and DO levels following magnetic exposure.

Furthermore, Ebrahim and Azab (2017) suggested that exposure of water to magnetic fields might modify hydrogen bonding and water cluster dynamics, thereby reducing surface tension and hypothetically increasing fluid permeability. In theory, such physical modifications could potentially facilitate nutrient dissolution and passive fluid dynamics across the gastrointestinal tract. However, it should be noted that physicochemical alterations in bulk water properties do not directly equate to immediate biological effects at the cellular level; rather, reduced surface tension may indirectly interact with membrane interfaces to support nutrient transport. Additionally, the elevated DO concentration is speculatively suggested to support an aerobic micro-environment near the mucosal interface or modestly influence ruminal fermentation kinetics.

However, because the physicochemical attributes presented in Table 2 reflect descriptive characterizations of the water treatments prior to the animal trial rather than experimentally tested response variables, these proposed cellular and metabolic pathways remain speculative. Whether these differences in water traits directly drive improvements in digestive activity, nutrient utilization, or metabolic efficiency in lambs cannot be conclusively confirmed from the present data and warrants further controlled cellular and physiological investigation.

### 4.2 Growth performance and nutrient digestibility

The significant improvements in final BW, ADG, and DMI observed in CA-supplemented lambs stem from specific biochemical and physiological actions of CA. As a phenolic organic acid, CA exhibits antimicrobial activity by disrupting bacterial membrane integrity and intracellular pH homeostasis, thereby suppressing pathogenic microorganisms and contributing to a more favorable ruminal and intestinal microbial ecosystem that supports fibrolytic and amylolytic bacteria (Varel and Jung, 1986; Peña-Torres et al., 2019; Tian et al., 2025; Orzuna-Orzuna et al., 2023). Furthermore, CA stimulates the secretion of endogenous digestive enzymes, including pancreatic lipase and trypsin, which enhance nutrient breakdown. This biochemical optimization explains the observed increase in DMD, OMD, CPD, and NDFD values. These findings align with Orzuna-Orzuna et al. (2023) and Peña-Torres et al. (2019), who reported that HAs improve performance through enzymatic and microbial modulation.

The positive effects of MW on digestibility may be related to biophysical modifications of water structure. Exposure of water to magnetic fields has been proposed to disrupt intra-cluster hydrogen bonds, leading to the breakdown of larger water clusters into smaller ones. However, contrary to the expected reduction in surface tension, experimental data demonstrate that surface tension can actually increase (from 72.27 to 75.50 mN/m), along with viscosity and enthalpy of vaporization. This increase has been attributed to the strengthening of inter-cluster hydrogen bonds in the newly formed smaller clusters, resulting from competition between the two hydrogen-bond networks (Toledo et al., 2008). These structural rearrangements may influence the solubility of dietary nutrients and could alter cellular membrane permeability in the gastrointestinal epithelium, thereby facilitating passive and active nutrient transport. Additionally, the elevated DO concentration in MW may enhance the metabolic efficiency of epithelial cells and digestive enzyme activity (Minoretti and Emanuele, 2024; Abd-El Azeem et al., 2025). These proposed biophysical mechanisms are consistent with previous observations in ruminants reported by Yacout et al. (2015) and Abdullaziz et al. (2022).

Although the combined administration of CA and MW yielded the highest numerical values for growth and digestibility, no statistically significant CA × MW interactions were detected. While this pattern suggests an additive physiological response, the lack of statistical interaction should be interpreted with caution, as the limited sample size (n = 6) may have restricted the statistical power required to detect subtle interactive effects.

Dietary CA supplementation significantly reduced total VFA concentration and acetate proportion while increasing propionate proportion and lowering the A:P ratio. Mechanistically, CA may modulate rumen microbial ecology through its selective antimicrobial properties (Annuur et al., 2024), altering microbial community composition and fermentation patterns toward greater efficiency (Ahmed et al., 2024; Chinnamani et al., 2024; Keum et al., 2024). The shift toward propionate is metabolically advantageous, as propionate serves as the primary substrate for hepatic gluconeogenesis, yielding greater net energy for lamb growth. The reduction in total VFA concentration might not necessarily reflect microbial inhibition, but could hypothetically be linked to accelerated ruminal absorption or altered epithelial transport kinetics, as suggested by Jiang et al. (2023).

The influence of MW on ruminal fermentation, marked by increased propionate and a reduced A:P ratio, might be speculatively attributed to subtle changes in the physicochemical dynamics or oxidation-reduction potential of the ruminal fluid. It is hypothesized that elevated DO levels and altered hydrogen bonding in MW could potentially influence liquid phase dynamics or microbial micro-environments in the rumen, thereby supporting propionate-yielding pathways. Similar fermentation shifts were reported by Al-Hafez et al. (2013). However, as direct measurements of ruminal microbial populations and epithelial transport kinetics were not performed in the present study, these mechanistic pathways remain theoretical and warrant further microbiological investigation.

### 4.3 Antioxidant status and inflammatory response

The marked enhancement in antioxidant biomarkers, evidenced by elevated SOD, GPx, and TAC along with reduced MDA, confirms the potent systemic antioxidant response to CA supplementation. Biologically, the structural features of CA, particularly its conjugated aromatic ring and α,β-unsaturated carboxylic acid moiety, facilitate hydrogen atom and electron donation, enabling free-radical scavenging and inhibition of lipid peroxidation chain reactions. (Freitas et al., 2024; Jomova et al., 2024).

In the literature, such antioxidant responses are frequently hypothesized to be mediated via the Nrf2 signaling pathway, where CA is suggested to promote Nrf2 nuclear translocation and upregulate genes encoding endogenous antioxidant enzymes (Yazdi et al., 2023). However, as Nrf2 nuclear translocation and downstream gene expressions were not directly quantified in the present trial, this molecular pathway remains a proposed mechanism requiring transcriptomic confirmation.

Concurrently, MW administration increased SOD, TAC, and GPx activities while decreasing MDA concentration, supporting earlier observations by Abdullaziz et al. (2022). It has been suggested that physical modifications in MW might influence cellular hydration dynamics or intracellular oxidative balance. However, as mitochondrial function, membrane potential, and cellular oxygen utilization were not directly evaluated in the present study, these physical mechanisms remain proposed hypotheses that require further targeted cellular studies.

Regarding the inflammatory response, the observed reduction in serum TNF-α concentration in CA-treated lambs is consistent with the recognized anti-inflammatory effects of CA. Mechanistically, the downregulation of TNF-α is hypothesized to involve the suppression of NF-κB activation and its nuclear translocation (Freitas et al., 2024). While our serum TNF-α data align well with these established anti-inflammatory patterns, direct intracellular and gene expression assays were not performed, and thus further molecular studies are required to definitively confirm the precise upstream signaling cascades operating in lambs.

### 4.4 Meat quality

Lower postmortem meat pH observed in treated lambs reflects normal postmortem anaerobic glycolysis kinetics, with all values remaining within the desirable range for lamb meat quality. The marked improvement in WHC and reduction in TBARS values suggest enhanced oxidative stability and structural integrity of muscle tissues during refrigerated storage. HAs derivatives, such as CA, are reported to scavenge free radicals and potentially protect membrane lipids from oxidation (Li et al., 2022).

Meat color traits were also influenced by the experimental treatments. Redness (a*) and chroma values were significantly affected, with significant (CA × MW) interactions indicating that the effect of CA supplementation on meat color stability depended on the level of water magnetization applied. Therefore, rather than assuming a generalized additive benefit, the combined response appears to be driven by specific interaction patterns between dietary CA and MW treatments. Furthermore, while subtle statistical tendencies (*P* < 0.10) were observed for WHC and LD area, these trends should be interpreted with caution and do not represent conclusive evidence of synergistic action. Overall, the preserved meat redness and lower TBARS values observed in specific combination groups may be associated with an enhanced systemic antioxidant status, which can reduce postmortem myoglobin oxidation and lipid peroxidation, thereby improving meat color and oxidative stability (Falowo et al., 2014; Domínguez et al., 2019).

### 4.5 Blood biochemical parameters

Circulating blood metabolites were determined to evaluate systemic physiological responses and hepatic lipid metabolism associated with dietary supplementation. The significant reductions in serum cholesterol and LDL concentrations, alongside the elevation in HDL, reflect hypolipidemic responses in CA-treated lambs. In monogastric models and cell studies, HAs have been reported to downregulate HMG-CoA reductase activity and act as potential ligands for PPAR-α, thereby modulating hepatic lipid synthesis and lipoprotein clearance (Peña-Torres et al., 2019; Wang et al., 2025). However, because ruminants possess distinct lipid metabolic pathways, which are characterized by extensive ruminal bio-hydrogenation of dietary fats and limited hepatic de novo lipogenesis compared with non-ruminants, these monogastric molecular mechanisms should be extrapolated to lambs with caution. The observed hypolipidemic effects in the present study may also involve altered ruminal VFA production or modified intestinal lipid absorption kinetics rather than direct hepatic enzyme inhibition alone.

A highly significant interaction between CA and MW (C × W; *P* < 0.001) was detected for cholesterol, HDL, and LDL. Pairwise comparisons revealed that this hypolipidemic action was specific to the (C1W0) group (CA without MW) and did not represent a generalized main effect across all treatment combinations. This isolated response suggests that under standard drinking water conditions, CA effectively modulates lipoprotein processing in lambs. Interestingly, when combined with MW (C1W1 and C1W2), this lipid-modulating effect was attenuated, returning concentrations to levels comparable to the control. This blunting effect may stem from potential physicochemical interactions between altered mineral cluster structures in MW and phenolic absorption dynamics, or altered ruminal microbial bio-hydrogenation patterns that merit further mechanistic investigation.

In contrast, circulating levels of glucose, BUN, TG, AST, ALT, and hematological indices remained within normal physiological ranges across all groups. The stability of hepatic transaminases and leukocyte profiles confirms that neither CA nor MW caused cytotoxic, hepatotoxic, or systemic inflammatory stress, verifying the biological safety of these dietary treatments under the experimental conditions.

### 4.6 Mechanisms underlying combined effects of cinnamic acid and magnetized water

The favorable physiological responses resulting from combined CA and MW administration likely stem from complementary biophysical and biochemical pathways. Experimental measurements indicate that magnetic exposure can alter hydrogen-bonding competition and reduce surface tension (Toledo et al., 2008), while reducing water molecular cluster sizes and increasing negative zeta potential, thereby modestly altering fluid dynamics and hypothetically enhancing cellular membrane transport efficiency. This physical alteration may promote the solubility, dispersion, and intestinal mucosal absorption of CA, thereby supporting its systemic bioavailability.

Concurrently, CA is hypothesized to exert intracellular antioxidant and anti-inflammatory functions potentially mediated via Nrf2 activation and NF-κB inhibition, while MW provides elevated DO that may support mitochondrial oxidative phosphorylation and epithelial metabolic activity. Furthermore, both treatments are suggested to exert localized synergistic actions in the digestive tract by optimizing ruminal fluid dynamics, enhancing fibrolytic bacterial activity, and stabilizing the mucosal barrier. Rather than operating through a single direct chemical reaction between CA and MW, the combined benefits may reflect complementary physiological effects, whereby physical transport facilitation by MW complements the metabolic actions of CA. However, because direct cellular uptake, membrane potential, and molecular signaling cascades were not quantified in the present trial, these combined pathways remain proposed working hypotheses that require further transcriptomic and cell-culture validation.

### 4.7 Study limitations and practical implications

Although the present study provides novel insights into the combined nutritional strategy of dietary trans-cinnamic acid supplementation and MW in fattening lambs, some limitations should be acknowledged.

First, a primary limitation of this study is the sample size of six animals per treatment group and the lack of an a priori statistical power analysis. While n = 6 was sufficient to detect robust main effects of CA and MW across several performance, digestive, and carcass parameters, the statistical power to detect small-to-moderate treatment responses, particularly two-way interaction effects (C × W), was inherently limited. Consequently, non-significant interactions or non-significant main effects should not be unequivocally construed as a lack of biological effect, but rather interpreted with appropriate caution.

Second, while the physiological and biochemical responses suggest additive health and antioxidative benefits, the underlying subcellular mechanisms (such as specific gene expression related to ruminal transport or muscle lipid peroxidation) were inferred from biochemical markers rather than direct transcriptomic analyses. Therefore, future studies utilizing larger sample sizes, broader dose-response ranges, and molecular evaluations are recommended to further confirm these findings and validate their practical and commercial applicability under extensive feedlot conditions.

## 5 Conclusions

Dietary supplementation with cinnamic acid (400 mg/kg DM) and magnetized water (4000 or 6000 Gauss) individually improved nutrient digestibility, shifted ruminal fermentation toward propionate, enhanced systemic antioxidant capacity, and improved meat color stability in fattening lambs. However, significant interaction effects were confined to specific lipid fractions and meat color parameters, whereas most other parameters showed no significant interaction. Given the limited sample size (n = 6) and lack of an a priori power analysis, the absence of statistical interactions in certain traits should be interpreted cautiously rather than as definitive evidence of independence. Overall, both interventions appear to be safe and promising nutritional strategies for improving lamb productivity and meat quality under the conditions of the present study, although future large-scale studies with greater statistical power are required to confirm these findings and fully elucidate potential synergistic effects.

## Ethics approval and consent to participate

All experimental procedures involving animals were approved by the Institutional Animal Care and Use Committee of the University of Torbat-e Jam (Approval No. 2231) and conducted in accordance with the National Research Council (NRC, 2007) guidelines for the care and use of laboratory animals.

## Consent for publication

Not applicable.

## Availability of data and materials

The data that support the findings of this study are available from the corresponding author upon reasonable request.

## Statement for studies in animals

All animal procedures were conducted in compliance with the guidelines established by the Iranian Council of Animal Care (1995) and received approval from the Institutional Animal Care and Use Committee at the University of Torbat-e Jam, Iran. Rumen fluid was collected from three ruminally fistulated Moghani male sheep, as detailed in the Materials and Methods section. Every effort was taken to reduce any potential pain or distress to the animals. Throughout the study, the animals were kept under standard management conditions, with unrestricted access to water and a balanced maintenance diet.

## Funding

This research was financially supported by the University of Torbat-e Jam.

## CRediT authorship contribution statement

**Mohsen Kazemi:** Writing – review & editing, Writing – original draft, Visualization, Validation, Supervision, Software, Resources, Project administration, Methodology, Investigation, Funding acquisition, Formal analysis, Data curation, Conceptualization. **Reza Valizadeh:** Writing – review & editing, Writing – original draft, Visualization, Validation, Methodology, Investigation, Data curation. **Abbas Khashei-Siuki:** Writing – review & editing, Writing – original draft, Validation, Methodology, Investigation, Data curation.

## Declaration of competing interest

The authors declare that they have no known competing financial interests or personal relationships that could have appeared to influence the work reported in this paper.
